# Um Paciente, Duas Cardiomiopatias

**DOI:** 10.36660/abc.20190853

**Published:** 2020-09-18

**Authors:** Christopher Strong, Pedro Freitas, António Ferreira, Gustavo Rodrigues, Miguel Mendes

**Affiliations:** Hospital de Santa Cruz Centro Hospitalar Lisboa Ocidental Lisboa Portugal Serviço de Cardiologia, Hospital de Santa Cruz, Centro Hospitalar Lisboa Ocidental, Lisboa - Portugal

**Keywords:** Displasia Arritmogênica Ventricular Direita, Cardiomiopatia Hipertrófica, Taquicardia Ventricular, Exercício, Síncope, Cardioversão Elétrica, Mutagênese

**Figura 1:**
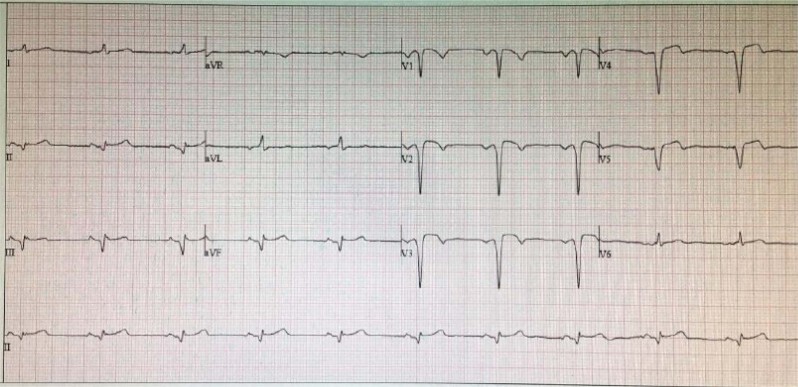
– Eetrocardiograma após cardioversão elétrica.

**Figura 2:**
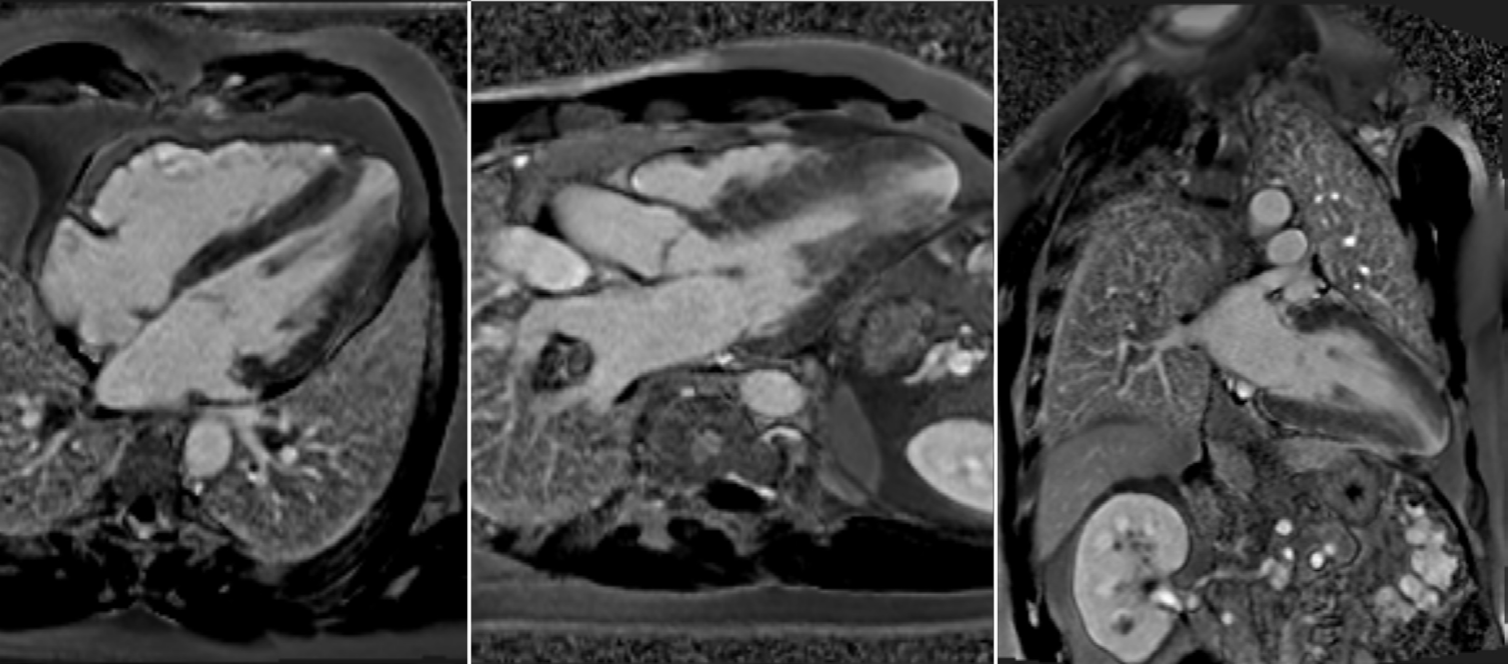
– Ressonância magnética cardíaca — realce tardio no ápice do ventrículo esquerdo e parede livre do ventrículo direito.

Paciente do sexo masculino, com 50 anos de idade, histórico de hipertensão bem controlada e sem histórico pessoal ou familiar conhecido de doença cardíaca, apresentou dor torácica seguida de síncope durante atividade física extenuante. O eletrocardiograma mostrou taquicardia ventricular monomórfica sustentada. Realizou-se cardioversão elétrica, com conversão para ritmo sinusal com ondas Q nas derivações V2-5 e III-aVF (Figura 1). A ecocardiografia transtorácica (ETT) mostrou hipertrofia ventricular esquerda (VE) assimétrica, fração de ejeção (FE) preservada com acinesia apical e ventrículo direito (VD) hipertrabeculado. A angiografia coronariana não mostrou lesões significativas. A ressonância magnética cardíaca confirmou os achados da ETT, mostrando espessura máxima da parede do VE de 17 mm no septo interventricular (Figura S1 — material complementar), aneurisma apical do VE e inserção anormal dos músculos papilares. O lado direito destacou-se pelas paredes livres e inferiores hipertrabeculadas e hipocinéticas, e VD levemente dilatado com FE reduzida (33%). Além de apresentar realce tardio transmural no ápice do VE, havia também realce tardio nodular na junção VE/VD e nas paredes basais livres e inferiores do VD (Figura 2; Vídeos 1–2; Figura S2 do material suplementar). A análise genética encontrou mutação heterozigótica no gene PKP2 (p.Thr50Serfs*61), uma variante patogênica associada à cardiomiopatia arritmogênica do VD.^1^ No entanto, nenhuma mutação conhecida nos genes relacionados à cardiomiopatia hipertrófica (CMH) foi encontrada em um painel de 204 genes, com 118 relacionados à CMH. Nenhuma associação foi firmemente estabelecida entre as mutações no gene PKP2 e CMH.^2^ A sobreposição entre os fenótipos genéticos e da cardiomiopatia é um fenômeno bem conhecido.^3^ Partimos da hipótese de que esse paciente tenha uma mutação adicional no gene CMH desconhecida ou, menos provavelmente, uma expressão fenotípica de duas cardiomiopatias diferentes no contexto de uma mutação do gene PKP2.

**Vídeo 1:** – Ressonância magnética cardíaca de 4 câmaras (à esquerda) e 3 câmaras (à direita). URL: http://abccardiol.org/supplementary-material/2020/11503/2019-0853_video01.mp4

**Vídeo 2:** – Ressonância magnética cardíaca — vista do trato de saída e entrada do ventrículo direito (à esquerda) e vista de eixo longo do trato de saída do ventrículo direito (à direita). URL: http://abccardiol.org/supplementary-material/2020/11503/2019-0853_video02.mp4
