# Supplementary material for: Um Paciente, Duas Cardiomiopatias
Source: Arq Bras Cardiol. 2020 Sep 18;115(3):584–6. [Article in Portuguese] doi: 10.36660/abc.20190853 (PMC9363104; doi:10.36660/abc.20190853)
Supplement: Translation

# Translation: One Patient, Two Cardiomyopathies

**Authors:** Christopher Strong, Pedro Freitas, António Ferreira, Gustavo Rodrigues, Miguel Mendes

**Affiliations:** Portugal Cardiology Department, Hospital de Santa Cruz, Centro Hospitalar Lisboa Ocidental, Lisbon - Portugal

**Keywords:** Arrhythmogenic Right Ventricular Dysplasia, Cardiomyopathy, Hypertrophic, Tachycardia, Ventricular, Exercise, Syncope, Electric Countershock, Mutagenesis

A 50-year-old male patient, with history of well-controlled hypertension and no known personal or family history of cardiac disease, presented with chest pain followed by syncope during strenuous physical activity. The electrocardiogram showed sustained monomorphic ventricular tachycardia. Electrical cardioversion was performed, with conversion to sinus rhythm with Q waves on leads V2-5 and III-aVF (Figure 1). Transthoracic echocardiography (TTE) showed asymmetric left ventricular (LV) hypertrophy, preserved ejection fraction (EF) with apical akinesia, and hypertrabeculated right ventricle (RV). Coronary angiography showed no significant lesions. Cardiac MRI confirmed the TTE findings, showing a maximum LV wall thickness of 17 mm at the interventricular septum (Figure S1 — supplementary material), apical LV aneurysm and abnormal insertion of papillary muscles. The right side was remarkable for hypertrabeculated and hypokinetic free and inferior walls, and mildly dilated RV with reduced EF (33%). Besides having transmural delayed enhancement (DE) on the LV apex, there was also nodular DE on the LV/RV junction and on the free and inferior basal walls of the RV (Figure 2; Videos 1–2; Figure S2 of supplementary material). Genetic analysis found a heterozygous mutation on the PKP2 gene (p.Thr50Serfs*61), a pathogenic variant associated with arrhythmogenic RV cardiomyopathy.^1^ However, no known mutation on genes related to hypertrophic cardiomyopathy (HCM) were found on a panel of 204 genes, 118 related to HCM. No association has been firmly made between mutations on the PKP2 gene and HCM.^2^ The overlap between genetics and cardiomyopathy phenotypes is a well-known phenomenon.^3^ We hypothesize that this patient either had an additional unknown HCM gene mutation or, less likely, a phenotypic expression of two different cardiomyopathies in the context a PKP2 gene mutation.

**Figure 1:** – The electrocardiogram after electrical cardioversion.

**Figure 2:** – Cardiac magnetic resonance imaging — delayed enhancement on the left ventricular apex and free wall of the right ventricle.

**Vídeo 1:** – Cardiac MRI 4-chamber view (left) and 3-chamber view (right). URL: http://abccardiol.org/supplementary-material/2020/11503/2019-0853_video01.mp4

**Vídeo 2:** – Cardiac MRI — right ventricle inflow and outflow view (left) and right ventricle outflow tract long axis view (right). URL: http://abccardiol.org/supplementary-material/2020/11503/2019-0853_video02.mp4
